# Technology-enabled remote management of diabetes foot disease and potential for reduction in associated health costs: a pilot study

**DOI:** 10.1186/s13047-020-00444-6

**Published:** 2021-01-13

**Authors:** Fiona Main, Ania Zubala, Jane Gorman, Sandra Jones, Jenny Hall, David Macfarlane, Sandra MacRury

**Affiliations:** 1 Highland Diabetes Institute, Centre for Health Science, Inverness, IV2 3JH UK; 2 Division of Rural Health and Wellbeing, University of the Highlands and Islands, Inverness, IV2 3JH UK

**Keywords:** Diabetes, Foot disease, Technology-enabled, Pathway, Amputation, Economic analysis

## Abstract

Diabetes-related foot disease, particularly when associated with amputation, affects quality of life and has a significant impact on health care costs. A pilot study using enhanced technology to facilitate remote access and video conferencing from rural locations to the diabetes MDT through a new service pathway confirmed high levels of patient satisfaction with 89% of foot ulcers improved or stable and only two minor amputations. A health economic analysis suggested potential for significant cost savings if this was scaled up regionally. Further evaluation of an integrated pathway, impact on lower limb amputation rates and full health economic assessment is recommended.

## Background

**Fig. 1:**
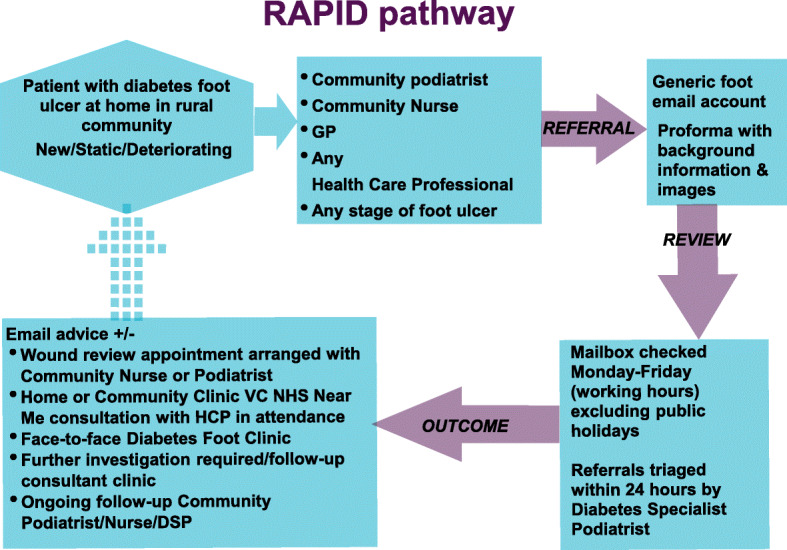
RAPID Pathway: process flow for community patient referral and triage to specialist diabetes foot services

Diabetes foot ulceration can significantly impact quality of life and up to 20% of infected foot ulcers will require an amputation [1]. Furthermore, it has been recognised that a diabetes foot ulcer and a lower extremity amputation are independent risk factors for premature death [1]. Based on NHS England data It is estimated that more than £80 million is spent on foot ulcers and amputations annually in Scotland alone [2]. Early access to the diabetes foot multi-disciplinary team (MDT) is associated with a reduction in amputations [3]. However, in the Scottish Highlands local audits highlighted that more than half of patients who subsequently underwent an amputation, were unknown to or had presented late to the diabetes specialist podiatrists / MDT. Accessing specialist services is challenging in rural areas and incurs significant excess travel distances and costs for patients. Building on our previously developed RAPID pathway (Fig. 1) to triage and manage diabetes foot ulceration in rural areas [4], we conducted a further pilot study that included an additional enhancement of the technology by incorporating ‘Direct Access’ software to enable remote access to clinical databases and a new video conference platform. We also developed an economic model to assess the potential value of the RAPID intervention project based on this pilot study.

## Methods

Two community podiatrists reviewed patients with diabetes foot ulcers in patients’ homes or local clinical settings in two defined rural areas over a six-month period using technology enabled care. A tablet device was used to capture images and, together with ‘Direct Access’ software, allowed remote access to a generic email account and clinical databases. An Omnirouter mini™ device aided connectivity through prioritisation of cellular networks. The tablet device was used for video-consultations, employing the NHS Near Me (Attend Anywhere) platform. This was facilitated by the community podiatrist with the patient in their own home. Patients were triaged according to the pathway shown in Fig. 1. Ulcer duration, healing rates, amputations, and patient experience were collected. An economic model was developed to assess the value delivered by the RAPID intervention versus costs attributable to the health, and care system for those without access, based on data from the pilot study and extrapolated to the wider area. Financial proxies were identified to quantify return on investment. Estimation of amputation rates was based on the local diabetes register (17,567) and 15% lifetime cumulative incidence of foot ulceration [1], giving an amputation rate of 9% in Highland region. The modelling relied on a series of assumptions around physical, psychological and social impact costs.

## Results

Thirty-one patients were referred for specialist advice using the pathway. Following triage, there were 110 community-based podiatry visits (range 1–10 visits/patient), including 45 successful video-consultations with the MDT. The average home appointment time was 45 min, with actual VC times of 6–14 min. There were six failed VC connections due to technology problems. In summary, a total of 55 ft ulcers were treated over the course of the study. A total of 89% of ulcers healed, improved, or remained stable with an improvement or healing noted in 27 (49.1%), including full healing in 18 wounds (33.2%) during the course of the project. Of the remainder, 22 (40.0%) ulcers were stable and four (7.3%) deteriorated. There were 2 (3.6%) minor amputations and no major amputations. Results are presented in Table 1. A high satisfaction level was reported by patients with the service pathway and technology, at 99 and 96% respectively.

**Table 1:** Patient characteristics and ulcer outcomes

Total participants (***n***= 31)	Male ***n***=18	Female ***n***= 13
**Age (yr)**	**71**	**74**
**(Mean + range)**	**51–84**	**53–89**
**Location of consultation (%) (*n*=110)**		
**-Home**	**41.8**	**26.3**
**-Local clinic**	**13.6**	**8.18**
**-Care home**	**4.5**	**0.9**
**-Not recorded**	**1.8**	**2.7**
**Number of ulcers per patient (%) (*n*=31)**		
-1	**29.0**	**25.8**
-2	**9.7**	**9.7**
-3	**16.1**	**3.2**
-4	**0**	**0**
-5	**3.2**	**3.2**
**Outcomes of ulcers (%**) **(*n*=55)**		
**Improved and healing**	**38.2**	**10.9**
**- Complete healing**	**25.5**	**7.3**
**- Improved**	**12.7**	**3.6**
**Stable**	**18.1**	**21.8**
**Deteriorating**	**3.6**	**3.6**
**Minor toe amputations**	**0**	**3.6**

Based on foot ulcer and minor amputation data within our area the economic analysis hypothetical cost valuation suggested potential cost savings of £138,820 vs £252,124 without RAPID, giving a return on investment ratio of 1:1.8 meaning £1.80 saved for every £1 invested. This is likely to be an underestimate of cost saving attributed to major amputations and does not consider improved quality of life or wider social cost savings.

There are several limitations: predominantly that it was pilot study in a relatively small group of individuals with no comparative control group. Accordingly, the limited number of minor and no major amputations over the short time-period may have been a chance finding or related to referral bias. The economic analysis was based on a number of assumptions and evidence-based research and related to a small group intervention.

## Conclusion

Remote patient management with a shift away from hospital-based care, to community and home-based care and use of telemedicine and/or VC is becoming increasingly common, particularly in relation the COVID-19 pandemic. The use of VC platforms has also been shown to be effective in screening for infection and wound-based assessment in people with diabetes [5]. The new service pathway confirmed that patients with a diabetes foot ulcer can be triaged within 24 h, ensuring timely access to a specialist foot team. There was a high level of patient satisfaction. An embedded pathway and technology solution including remote consultation can permit early intervention in patients with foot ulcers, with the potential to reduce amputations. The economic assessment was modelled on a small number of patients; an expanded assessment at regional level linked to minor and major amputations numbers will allow improvement in the cost valuation model.

## Data Availability

Non-applicable.

## Abbreviations

MDT: Multidisciplinary team
RAPID: Reducing Amputations in People with Diabetes
NHS: National Health Service
VC: Video-consultation
